# Impact of Media and Induction Strategy on Physicochemical Characteristics and Immunogenicity of Recombinant fHbp-PorA Chimeric Protein: A Promising Meningococcal B Vaccine Candidate Produced in *Escherichia coli*

**DOI:** 10.3390/vaccines14050382

**Published:** 2026-04-24

**Authors:** Annamraju Aswini, Annamraju D. Sarma, Ashish B. Deshpande, Yogesh C. Padwal, Vinay V. Gavade, Sambhaji S. Pisal, Selvan Ravindran

**Affiliations:** 1 Serum Institute of India Pvt. Ltd., Hadapsar, Pune 411028, India; phdgrad.annamraju.aswini@siu.edu.in (A.A.); ad.sarma@seruminstitute.com (A.D.S.); ashish.deshpande@seruminstitute.com (A.B.D.); yogesh.padwal@seruminstitute.com (Y.C.P.); vinay.gavade@seruminstitute.com (V.V.G.); sspisal@seruminstitute.com (S.S.P.); 2 Symbiosis School of Biological Sciences (SSBS), Faculty of Medical and Health Sciences, Symbiosis International (Deemed University), Lavale, Pune 412115, India

**Keywords:** Meningococcal B chimeric protein, recombinant vaccine, fHbp-PorA, *Escherichia coli*, media optimization, induction strategy, immunogenicity

## Abstract

Background/Objective: Apart from the attributes such as cost, yields and consistency that define the feasibility of a manufacturing process, physicochemical and immunological quality traits equally signify the functionality of a biological product. The present study investigates one such promising Meningococcal B vaccine candidate, a chimeric fHbp-PorA protein in *Escherichia coli.* Methods: The chimeric fHbp–PorA protein, expressed with an N-terminal tag of HIS-MBP-TEV was produced in a 10 L fermenter under two different media and induction strategies: chemically defined (CD) media with lactose induction and complex media (CM) with galactose-mediated autoinduction. Comparative analysis was carried out between the two approaches for cell growth, protein expression, and purification, and the final chimeric proteins were characterized to evaluate for their biochemical, structural, *in vitro* and *in vivo* immunochemical properties. Results: Growth in the CD media resulted in several-fold-higher biomass compared to that in CM media in a short cultivation time; however, more than a third of the expressed protein remained in an insoluble state. Meanwhile, almost all of the expressed protein with CM media was recovered in soluble form. Moreover, purification of the unprocessed tagged protein and recovery of chimeric protein (tag removed) resulted in 75% greater yield in CM media when compared to CD media. The final chimeric proteins obtained from each medium varied significantly in their physicochemical characteristics, including their epitope projection and CD spectra. The results of *in vivo* animal immunogenicity response also showed higher serum bactericidal activity associated with chimeric protein obtained from CM media compared to CD media. Conclusions: The outcomes demonstrate that complex media with galactose-induced expression not only show higher productivity but also exhibit superior quality attributes, qualifying their reliable use in the manufacturing process of this promising vaccine candidate.

## 1. Introduction

*Neisseria meningitidis* is an obligate human pathogen that resides in the nasopharyngeal tract. Globally, 6 of the 13 serogroups are classified under aggressive types that cause invasive meningococcal disease, leading to considerable morbidity and mortality [1,2]. Prophylactic glycoconjugate vaccine against five of these serogroups has been successfully introduced and used in various countries/regions. However, an effective glycoconjugate for meningococcal serogroup B remains elusive owing to its weak immunogenic capsular polysaccharide (CPS) and due to its similarity to human neuronal cells, leading to possible autoimmune damage [3,4].

Pioneering work by Masignani et al. (2019) [5] led to the discovery of a range of vaccine antigens with potential to elicit a protective immune response against the Men B serogroup. Factor H binding protein (fHbp) is one such vaccine antigen, which is a surface-exposed meningococcal lipoprotein. As the name suggests, it binds to human complement factor H (CfH) and prevents the activation of the complement pathway, helping the bacteria to evade the immune system. Based upon its expression and antigenic variability, the fHbp elicits antibodies with bactericidal activity predominantly in a subfamily/variant-specific manner. Three main variants have been identified (subfamily A/variant 2 and 3 and subfamily B/variant 1), and amino acid sequence has revealed several subvariants with extensive genetic and antigenic variabilities [6]. It has been reported that a single fHbp variant can cross-protect strain/subvariants that share at least 80% sequence similarity but offers no or low cross-protection against strains/subvariants with different fHbp that show low sequence identity. In this regard, variants 2 and 3 are immunologically related and elicit antibodies that cross-protect across this subfamily but do not offer protection against subfamily B/variant 1 [7]. As the immune response against fHbp elicits antibodies with complement-mediated bactericidal activity, it is included in both the present-day licensed vaccines, namely recombinant 4CMenB (Bexsero) and MenB-fHbp (Trumenba) [7,8]. Interestingly, in a parallel study, it was reported that interaction between fHbp and CfH could reduce the number of protective epitopes [9], and it was later shown that non-CfH binding fHbp mutants generated using site-directed mutagenesis, when tested in transgenic mice, elicited higher immunogenic response compared to wild-type fHbp of the same variant [10].

More recently, a new concept of a multi-antigen in a single protein has been developed at the University of Oxford [11] wherein a chimeric protein was generated using fHbp as a molecular scaffold. A portion of another potential immunogenic antigen, namely Porin A (PorA), was inserted at specific sites, resulting in loss of the CfH binding property by the chimeric molecule. When presented to animals, the chimeric protein elicited strong immune response towards both the fHbp and PorA portions of the molecule and showed serum bactericidal activity against several clinical isolates. It is anticipated that by employing the current epidemiologic data, a combination vaccine using multiple chimeric molecules can be developed that not only increase the coverage but also reduce the potential risk of vaccine escape.

To further take this concept of a chimeric protein-based vaccine for preclinical and clinical trials, we recently reported the optimization and validation of an autoinduction medium for growth and expression of a recombinant His-MBP-tagged fHbp-PorA chimeric protein [12]. The work describes a consistent and scalable process for achieving high yields of soluble tagged chimeric protein. However, the previous report does not show any indication of the final state of the chimeric protein (tag removed), its impurity profile, solubility, or any immunological characteristics. It is therefore imperative to establish these quality attributes before its intended use as an effective vaccine.

The choice of growth medium and its optimization is central to the production of high-quality recombinant protein, as this directly impacts the structural integrity of the expressed protein by significantly altering the growth rate, metabolic dynamics and protein folding environment of the cell. In order to maintain a balance between productivity and growth dynamics, implementing strategies to overcome undesirable limitations caused by media components, process parameters, and inducer/induction conditions are critical for process development.

With this objective to understand the effect of process-related conditions and constraints on the productivity, yield, solubility, functionality, safety and immunogenicity of the chimeric protein, we report an evaluation of two production processes that differ in terms of growth media, growth conditions and inducer and induction settings. The first relies on defined media using glucose or glycerol as carbon sources for high biomass accumulation prior to induction by lactose, typically similar to the auto-induction method described by Studier (2005) [13]. The second follows our previously reported optimized process with complex media containing yeast extract, soy phytone and galactose, which synergistically and simultaneously allows high growth and expression. The results significantly justify that the chimeric protein produced from the autoinduction process using CM media is superior in terms of high specific productivity and also with respect to its higher-quality attributes, including solubility, safety and immunogenicity.

## 2. Materials and Methods

### 2.1. Bacterial Host Strain

The recombinant *Escherichia coli* B834 (DE3) strain (Sigma-Aldrich, Burlington, MA, USA) with the pET-28a (+) plasmid (EMD Millipore, Temecula, CA, USA) expression vector harboring the chimeric fHbp-PorA gene was obtained from Oxford University. The details of the cloning methodology are described in our previous report [12].

A cryoprotective freezing media comprising Luria Bertani (LB) broth with 25% (*v*/*v*) glycerol was used to prepare a research cell bank (RCB). These were stored at −80 °C to maintain cell viability and genetic stability.

### 2.2. Media and Fermentation Processes

#### 2.2.1. Fermentation in Chemically Defined Media

The chemically defined media used in this process was named M9 modified minimal media and consisted of glucose (10 g/L), K_2_HPO_4_ (15 g/L), KH_2_PO_4_ (7.5 g/L), citric acid (2.0 g/L), (NH_4_)_2_SO_4_ (2.5 g/L), MgSO_4_·7H_2_O (1.4 g/L), L-Methionine (750 mg/L), thiamine hydrochloride (10 mg/L) and 10 mL of trace elements solution (i.e., FeSO_4_·7H_2_O (2.8 g/L), MnSO_4_·H_2_O (2.68 g/L), CoSO_4_·6H_2_O (1.42 g/L), CaCl_2_·2H_2_O (1.5 g/L), CuSO_4_·5H_2_O (0.18 g/L), ZnSO_4_·7H_2_O (0.3 g/L), dissolved in 1 L of 1 M HCl).

One vial of RCB was inoculated into 30 mL M9 minimal media in a 150 mL shake flask and incubated at 37 °C and 250 rpm for 4–6 h (Flask 1). Once the culture reached optical density at 590 nm (OD_590_) 2.5–3.5, it was expanded under the same conditions by subculturing in fresh media to a total of 300 mL and designated as the seed culture (Flask 2). The seed culture was then aseptically transferred to a Bioflo 120 Eppendorf 10 L fermenter containing 2.7 L of M9 medium. Subsequently, the cultivation was carried out using the following set process parameters: temperature of 37 °C, pH of 7.0, air flow of 3–5 SLPM, dissolved oxygen (DO) 35% and agitation speed of 200–1200 rpm. The pH during the process was maintained by using 6% Ortho-Phosphoric acid and 14% ammonia solution, respectively, and 10% Struktol was used as an antifoam. Culture growth was monitored by measuring OD_590_ at hourly intervals. After 5–6 h, when the initial glucose in the medium was depleted, a glucose feed (glucose (500 g/L), MgSO_4_·7H_2_O (24.6 g/L), thiamine hydrochloride (70 mg/L) and 15 mL of trace elements (same as above)) was introduced and continued for 3–4 h. When the OD_590_ reached 60–85, the glucose feed was discontinued and a glycerol feed (glycerol (500 g/L), MgSO_4_·7H_2_O (24.6 g/L), thiamine hydrochloride (70 mg/L) and 15 mL of trace elements (same as above)) was initiated. Simultaneously, the culture was induced by adding lactose (5–10 g/L). Induction was continued for 4–6 h until the OD_590_ reached saturation. After a couple more hours, the batch was harvested by centrifugation at 4700 rpm for 40 min at 4 °C using the Thermo Scientific Sorvall RC12BP+ refrigerated centrifuge (Thermo Scientific, Waltham, MA, USA). The harvested cell pellet was kept at −80 °C until further purification. The total run time of the process was approximately 14–16 h.

#### 2.2.2. Fermentation in Complex Autoinduction Media

A 10 L autoinduction cultivation was carried out in a Bioflo 120 Eppendorf 10 L fermenter (Eppendorf, Hamburg, Germany; Software version: BioCommand Revision 4.01.0006) using complex autoinduction media.

The media composition in this process basically consisted of three components, soy phytone, yeast extract, and galactose, which were present in varying amounts depending on the stage of cultivation. Revival Stage: One vial of the RCB was revived in 150 mL of seed media (yeast extract (5.0 g/L), 5.0 mM MgSO_4_·7H_2_O, and NaCl (2.0 g/L)) in an Erlenmeyer shake flask (Corning^®^, Corning, NY, USA). The seed culture was grown overnight at 25 °C under constant agitation at 220 rpm (Flask 1). Expansion Stage II: The overnight-grown culture was expanded by inoculating it directly into 700 mL of expansion media (soy phytone (10.0 g/L), yeast extract (5.0 g/L), 5.0 mM MgSO_4_·7H_2_O, NaCl (2.0 g/L) and glycerol (5.0 g/L) in an Erlenmeyer shake flask (Corning^®^). The cells were allowed to adapt for approximately 4–5 h at 25 °C under constant agitation at 220 rpm until the culture reached the optical density (OD at 590 nm) of 2.5–3.5 (Flask 2). Fermentation Stage: The matured expansion culture was then aseptically transferred to a fermenter holding 2.7 L of 10× complex autoinduction media (soy phytone (100 g/L), yeast extract (50 g/L), 25 mM MgSO_4_·7H_2_O, NaCl (10 g/L) and glycerol (25 g/L)). The cultivation was carried out using the following process parameters: temperature of 25 °C ± 2 °C, pH of 7.0 ± 0.2, air flow of 2 SLPM, dissolved oxygen (DO) 20 ± 2% and agitation speed of 250–1200 rpm. The culture pH was maintained using 6% Ortho-Phosphoric acid and 14% ammonia solution, and 10% Struktol was used as an antifoam. After 6–7 h, when the culture reached the early log phase, the feeding process was initiated using feed media (soy phytone (100 g/L), yeast extract (50 g/L), 25 mM MgSO_4_·7H_2_O, NaCl (10 g/L), glycerol (50 g/L) and 10 mM galactose) and continued until the culture showed early signs of decline (approximately 18–20 h). Subsequently, the cells were harvested using the Thermo Scientific Sorvall RC12BP+ refrigerated centrifuge and stored at −80 °C for further purification.

#### 2.2.3. Cell Lysis Using a Homogenizer

The expressed His-MBP-tagged chimeric protein from both of the above-mentioned processes was extracted and isolated from the harvested cells by subjecting them to lysis using a GEA Niro Soavi Lab Panda Homogenizer (GEA Niro Soavi, Parma, Italy). Approximately 75–100 g of cell mass obtained from each process was suspended in 2–3 L of lysis buffer (comprising 25 mM sodium phosphate buffer (pH 7.4), containing 100 mM NaCl and 5 mM imidazole) and subjected to 1000–1200 bar pressure to lyse the cells. The lysis process was repeated for 3–5 cycles, ensuring complete disruption of the cells. The lysate then was centrifuged using the Thermo Scientific Sorvall Lynx refrigerated centrifuge at 9000 rpm for 1 h at 4 °C. The clear supernatant containing the protein of interest was collected and filtered through a 0.45-micron membrane filter prior to purification.

#### 2.2.4. Purification of His-MBP-Tagged fHbp-PorA Protein by Metal Affinity Chromatography

Purification of the tagged chimeric proteins from both processes was performed using two Nickel Sepharose 6 Fast Flow columns (Cytiva, Uppsala, Sweden) connected to the ÄKTA Pure purification system (Cytiva (formaly GE Healthcare), Uppsala, Sweden; Software version: SULQETWPPTOUCH Version 1.1). Briefly, both the columns packed with metal affinity resin were washed with two column volumes (CVs) of water for injection (WFI) and subsequently charged with nickel ions using 100 mM nickel sulphate solution. This was followed by washing with WFI to remove unbound or loosely bound nickel ions, and equilibration was performed using 2–3 CV of equilibration buffer (25 mM sodium phosphate buffer (pH 7.4) containing 100 mM NaCl and 5 mM imidazole). Each tagged chimeric protein obtained from the respective processes was applied to the individually charged and equilibrated columns. Following the sample application, the non-specifically bound proteins were removed by sequential washes with buffers containing increasing concentrations of imidazole. The initial wash was performed with 25 mM sodium phosphate buffer (pH 7.4) containing 100 mM NaCl and 20 mM imidazole (5–6 CV), followed by a second wash using 25 mM sodium phosphate buffer (pH 7.4) containing 100 mM NaCl and 40 mM imidazole. Finally, each of the tagged protein was eluted using 25 mM sodium phosphate buffer (pH 7.4) containing 100 mM NaCl and 300 mM imidazole. Each eluted tagged protein was further subjected to micro-filtration using a 30 kDa tangential flow filtration (TFF) to remove any residues of imidazole, followed by concentration of the tagged protein. Finally, each protein was further dialyzed and buffer-exchanged using 12.5 mM sodium phosphate buffer (pH 7.4) on a 10 kDa cassette and filtered using a 0.2-micron filter before storing at 2–8 °C until further use.

#### 2.2.5. TEV Protease Mediated Removal of the His-MBP Tag from fHbp-porA Chimeric Protein

Presence of a Tobacco Etch Virus (TEV) protease site between the N-terminal His-MBP tag and the fHbp-PorA chimeric protein allows for easy removal of the tag by subjecting each of the purified tagged chimeric protein to proteolysis using this enzyme.

Briefly, each tagged protein was diluted to 10 mg/mL in 12.5 mM sodium phosphate buffer (pH 7.4), and 100 mM DTT was added to each tagged protein to reach a final concentration of 1.0 mM DTT. The TEV protease stocks, stored below −20 °C, were thawed and added to the tagged protein at a ratio of 1 part enzyme to 20 parts protein. The pH of the mixture was adjusted to 7.4 (if required), and the digestion was carried out by incubating the mixture in a shaker incubator with gentle agitation (40 rpm) at 30 °C for 16–18 h.

#### 2.2.6. Purification of Chimeric fHbp-PorA Protein

In order to obtain final chimeric protein devoid of the His-MBP tag and traces of TEV protease, the digest of each tagged chimeric protein was further subjected to purification by sequentially passing the digested proteins through two chromatographic steps, namely a HiTrap Capto^TM^ Q anion exchange column (Cytiva), followed by metal-affinity chromatography on a Nickel Sepharose 6 fast flow column (Cytiva).

Briefly, two HiTrap Capto^TM^ Q anion exchange columns were equilibrated with 2–3 CVs of 12.5 mM sodium phosphate buffer (pH 7.4) prior to the sample loading. Each digestion mixture of the chimeric proteins (filtered through 0.2 μm, adjusted to pH 7.4, and with conductivity of ~1.8–2.2 mS·cm^−1^) was applied to each column respectively, and the flow-through was collected. Following the application, each column was thoroughly washed with 3–4 column volumes of 12.5 mM sodium phosphate buffer (pH 7.4). The flow-through and wash fractions for each protein were separately pooled and concentrated, followed by diafiltration on a 5.0 kDa TFF cassette using 12.5 mM sodium phosphate buffer containing 100 mM NaCl and 5 mM imidazole. The chimeric protein was further filtered using a 0.2 μm filter and stored at 2–8 °C until the next purification step.

The second and final purification of chimeric proteins was achieved by metal-affinity chromatography on a Nickel Sepharose 6 Fast Flow column (Cytiva). Briefly, the column was initially washed with two CVs of water for injection (WFI), followed by activation using 250 mL of 100 mM nickel sulphate solution. Once charged, the column was washed with WFI and equilibrated using equilibration buffer (12.5 mM sodium phosphate buffer (pH 7.4), 100 mM NaCl and 5 mM imidazole). The chimeric protein obtained from the previous step of ion exchange chromatography was loaded onto the column, followed by a wash step of 3–4 CVs using the equilibration buffer. Elution of the bound protein was carried out using incremental concentrations of imidazole (30, 60 and 300 mM) in 12.5 mM sodium phosphate buffer (pH 7.4) containing 100 mM NaCl. Each elution step was carried out with at least 5–6 CV of buffer containing respective amounts of imidazole. More than 95% of the chimeric protein eluted in the first elution with buffer containing 30 mM imidazole. The eluted protein was filtered through a 0.2-micron filter and stored at 2–8 °C until use.

#### 2.2.7. Circular Dichroism Spectroscopy

CD spectroscopy was performed with the purified chimeric proteins from both the CD media and the CM media (0.2 mg/mL in 10 mM phosphate buffer) on a JASCO J-1500 spectropolarimeter (JASCO Inc., Easton, MD, USA) with a JASCO CTU-100 circulating thermostat unit to control the temperature of the optical cell chamber. The CD data was recorded using Spectra Manager Software, version 2.15, and analyzed using a CD multivariate SSE (secondary structure estimation; JASCO Inc., Easton, MD, USA; Software version: Spectra Manager version 2.15). To determine the % secondary structure, the CD spectrum was converted from mdeg. to molar ellipticity using molecular wt. 28.38 kDa with 267 amino acid residues and 0.1 cm of path length. For far-UV CD data acquisition, 10 mM phosphate buffer was used for baseline correction, and 0.2 mg/mL of bovine serum albumin was used for system suitability testing. Similarly, lysozyme (1.0 mg/mL) was used for the system suitability test near UV CD acquisition.

#### 2.2.8. Plasmid Isolation and Quantification

Plasmid DNA was isolated from recombinant *E. coli* cultures using the PureLink™ Plasmid Miniprep Kit (Invitrogen, Thermo Fisher Scientific, Waltham, MA, USA), following the protocol provided in the kit.

The plasmid DNA concentration and purity were assessed using a NanoDrop 1000 spectrophotometer (Thermo Fisher Scientific, USA), where 1–2 µL aliquot of each DNA sample was loaded onto the measurement pedestal, and absorbance readings were recorded at 260 nm and 280 nm. The DNA concentration (ng/µL) was calculated based on the A260 value, while the A260/A280 ratio was used to evaluate sample purity.

#### 2.2.9. fHbp- and PorA-Specific Enzyme-Linked Immunosorbent Assay (ELISA)

In order to assess the conformational integrity of the proteins expressed by both processes, each chimera was evaluated by ELISA using monoclonal antibodies directed towards specific surface-projected epitopes, namely Anti-meningococcal sero-subtype P1.4 (NIBSC, Potters Bar, UK, Cat.no: 02/148) for PorA loop detection and JAR-41 Anti-fHbp antibody (Absolute Antibody, cat.no: Ab03894-23.0), which binds to the fH binding site on the fHbp molecule.

PorA-specific ELISA: The antigen (fHbp-PorA chimera) was diluted in phosphate-buffered saline (PBS) to an appropriate final concentration (0.1–5.0 μg), and 50 µL of the diluted antigen was added to each well of the ELISA plate. For a blank control, the antigen was omitted, and 50 μL PBS alone was added to the well. The plate was incubated at 4 °C overnight. Using phosphate-buffered saline (PBS) containing 0.05% Tween20 (PBST), each well was washed three times and subsequently blocked by 4% bovine serum albumin in PBST, followed by incubation at 37 °C for 1 h. After washing the plate three times with PBST to remove the unbound BSA, 50 μL of primary mAb (PorA P1.4 mAb diluted 1/1000 in PBST) was added to each well, and the plate was incubated at 37 °C for 1 h. The unbound primary mAb was removed by washing the plate three times with PBST. Then, 50 μL of secondary antibody (Polyclonal goat anti-mouse HRP secondary antibody), diluted to a final concentration of 1/10,000 in PBST, was added, and the plate was incubated at 37 °C for 1 h. The unbound secondary antibody was removed by washing the plate three times with 300μL PBST, and the assay was developed by adding 100 μL of development reagent (prepared as a 1:1 mixture of solution A and solution B, as directed by the assay kit). Then, the plate was incubated at 25 °C/RT for 30 min, and the reaction was stopped by adding 50 μL of stop solution, which turned the reaction yellow. The absorbance of the color developed at 450 nm and 630 nm was read and recorded. Normalized absorbance (450 nm–630 nm) was used to plot graphs using GraPhpad PRISM version 10.6.1 software.

fHbp-Specific ELISA: Briefly, 96-well microtiter plates were coated with Anti-Factor H binding protein (JAR41) capture antibodies (Absolute Antibody) at 0.5 to 10 μg per well, diluted in phosphate-buffered saline (PBS, Gibco, Grand Island, NY, USA) containing 0.1% bovine serum albumin (BSA, Sigma-Aldrich, Burlington, MA, USA) and incubated for 18 h at 2–8 °C. This was followed by blocking the plate using 1% BSA in PBS per well and incubating it for 1.0 h at room temperature. Each chimera from both processes was serially diluted (0.5 mg/mL to 1 ng/mL) using 0.1% BSA in PBS, added to the plate and incubated at room temperature for 1.0 h. The detection antibody (Anti-meningococcal sero-subtype P1.4 (NIBSC, Cat.no: 02/148)) was diluted using 0.1% BSA in PBS (100-fold), and 100 µL was added to each well and incubated for 1.0 to 2.0 h at room temperature. After washing the plate thrice with PBST, the secondary detection antibody (Polyclonal goat anti-mouse HRP secondary antibody, Sigma) was diluted using 0.1% BSA in PBS (100,000-fold), and 100 µL was added to each well and incubated for 1.0 h at room temperature. The final reaction was initiated by adding 100 µL of tetramethylbenzidine (TMB, Sigma) substrate to each well and incubating for 30 min at room temperature, followed by stopping with the addition of 100 µL of 1N HCl to each well. Absorbance was read at 450/630 and plotted using GraPhpad PRISM software.

#### 2.2.10. Formulation and Animal Studies

First, 60 μg/mL of each chimeric protein obtained from both processes was adsorbed on 3.4 mg/mL of alhydrogel (aluminum hydroxide). The adsorbed proteins were suspended in 5 mM phosphate buffer, pH 7.2 ± 2, containing 50 mg/mL mannitol, 0.5 mg/mL polysorbate 20 and 5.0 mg/mL 2-phenoxyethanol. The suspension was stored at 2–8 °C until use. Individual formulations for each chimeric protein were evaluated for the following test parameters: total protein content, % protein adsorption, pH, zeta potential, osmolality and particle size distribution. Each sample was tested for its stability for at least one month at 2–8 °C. Each formulation was tested on in-house in-bred New Zealand Rabbits. Details of the immunization study design are presented in Table 1. All procedures involving animals were strictly performed as recommended by the Institutional Animal Ethics Committee, India.

#### 2.2.11. Serum Bactericidal Assay

The *N. meningitidis* serogroup B target strains (homologous strains: M08240157, M17240832; heterologous strains: M17240156, M16240272; stock cultures stored at −70 °C) were streaked for isolated colonies and incubated overnight at 37 °C with 5% CO_2_ on a CBAB-HB (Columbia Blood Agar Base with 5% Horse Blood) agar plate. The overnight-grown strain was subcultured by spreading cells over the center of another CBAB-HB agar plate and incubating it for 4 h at 37 °C with 5% CO_2_.

Using a sterile swab, the bacterial growth in the center of the CBAB-HB plate was swept once completely, and the bacteria were suspended in 5 mL of HBSS (Hanks Balanced Salt Solution containing MgCl_2_ and CaCl_2_, Gibco). Absorbance was measured on a spectrophotometer at 650 nm, and the suspension was adjusted to an OD of A_650_ = 0.1. A 1/10 dilution was done, followed by a 1/250 dilution to yield a final concentration of 6 to 10 × 10^4^ cells/well. A sterile 96-well microtiter plate was used to perform the assay, in which 20 μL of bactericidal buffer (HBSS+ or HBSS−) was added to all wells. Then, 20 μL of heat-inactivated serum was added to column 1. Serial dilutions of the test sera (two-fold) were performed across columns 1 to 9. Then, 10 μL of the working solution of bacteria was added (6 to 10 × 10^4^) to every well. Heat-inactivated complement (10 μL, 56 °C for 30 min) was added to all wells of column 11, and 10 μL of normal complement was added to columns 1 to 10. The plate was incubated for 1 h at 37 °C. Spotting was done using the tilt method, where 10 μL suspension from each well was spotted onto a blood agar plate. The plates were incubated overnight at 37 °C with 5% CO_2_. Colonies were counted the next day using a colony counter (Synbiosis-ProtoCOL3). The bactericidal titer for each unknown serum sample was expressed as the reciprocal serum dilution yielding ≥50% bacterial killing relative to the complement control wells. Titers that fell below the lower limit of detection were assigned a value of ‘2’ for calculation of the geometric mean titers (GMTs). Statistical analysis comparing titers obtained from the chimeric proteins produced in CD and CM media was performed using a paired *t*-test (statistical significance of *p* ≤ 0.05). GraphPad Prism version 10.6.1 software was used for statistical analysis.

## 3. Results and Discussion

### 3.1. Effects of Media, Cultivation and Induction Conditions on Expression and Productivity

Growth media, physical parameters (pH, temperature, dissolved oxygen) and the type of inducer used for culture cultivation significantly affect the metabolic rate, cellular environment and host stress response during recombinant protein expression in *E. coli* [14,15]. In order to understand the intricate effects of these factors on the cell growth, productivity, and quality of the fHbp-PorA chimeric protein, we cultured and expressed this protein in two different media, namely (1) chemically defined medium comprising the modified minimal medium supplemented with glucose, glycerol feed and lactose as an inducer at 37 °C, and (2) complex medium containing phytone, yeast extract, and galactose as an inducer at 25 °C, respectively.

**Figure 1:**
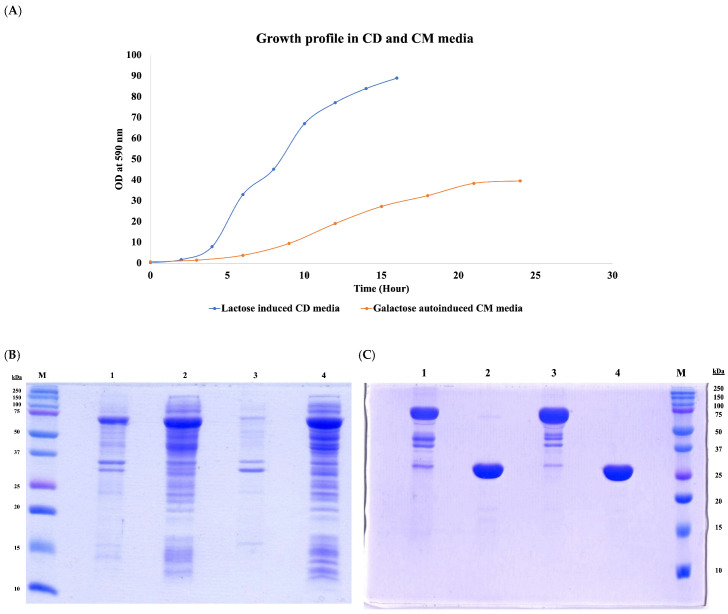
(**A**) Comparative fed-batch fermentation of lactose-induced CD media vs. galactose-induced autoinduction media. (**B**) Comparative SDS-PAGE analysis of expressed target protein in CD media using lactose induction (lane 1: insoluble fraction; lane 2: soluble fraction) and CM media with galactose autoinduction (lane 3: insoluble fraction; lane 4: soluble fraction). (**C**) Comparative SDS-PAGE analysis of purified tagged chimeric protein (lanes 1 and 3) and final chimeric protein without tag (lanes 2 and 4) from CD and CM media, respectively. The original SDS-PAGE figures can be found in Supplementary Materials.

Figure 1 clearly shows that the CD media significantly supported the accumulation of biomass, with harvest reaching 80–90 OD_590_ within 12–14 h. In contrast, the complex media took nearly 24 h to reach saturation, with a final harvest of 35–40 OD_590_. This was also reflected in the total biomass accumulated across both media (Table 2), with the CD media accumulating twice as much biomass as the CM media. However, major changes were observed with respect to the protein expression, specific productivity and impurity profile under both conditions. First, 30–40% of the expressed protein in the CD media appeared in the insoluble fraction, whereas nearly 95% of the expressed protein remained in the soluble fraction in the CM media (Figure 1B). This observation was also reflected in the subsequent purification of both the tagged protein and the final chimeric protein, where 70–75% increased specific productivity and yield were observed in the CM media compared to the CD media (Table 2). Second, major impurities, such as host cell proteins, DNA and endotoxins, were several folds lower in the chimeric protein obtained from the CM media compared to that obtained from the CD media (Table 2).

The production of recombinant proteins under the *lac* promoter using IPTG is a well-established method. Alternatively, Studier [13] introduced the concept of autoinduction, which is based on the function of the *lac* operon regulatory elements in mixtures of glucose, glycerol and lactose, where the host first undergoes a non-inducible phase (biomass accumulation) followed by an induction phase (product accumulation). These diauxic growth and induction conditions eliminate the need for continuous monitoring and reduce small changes in the inoculation volume and differences in the medium composition. Several variations of this phenomenon have recently been reported for a number of proteins as an alternative application in the production of recombinant protein [16].

**Figure 2:**
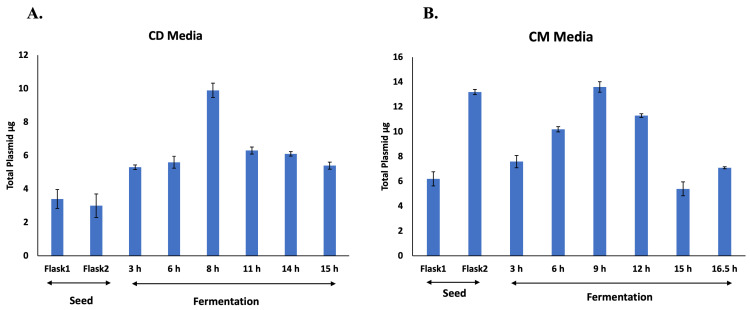
Comparative analysis of plasmid content during fermentation in lactose-induced CD media (**A**), vs. galactose-induced autoinduction CM media (**B**). The X-axis represents various stages of culture development or the hourly time points of bacterial culture during fermentation. The Y-axis represents the total plasmid content obtained from culture (cell mass normalized to 1.0 OD_590_) at various stages of culture development or the hourly time points of bacterial culture during fermentation.

The main purpose of this strategy is to optimally use the host resources specific to cell growth or the induction phase during fermentation. The CD media process described in this study is similar to the two-phase fermentation protocol suggested by Studier; it mainly relies on glucose, glycerol and other components (trace elements and vitamins such as thiamine and amino acids) for growth. When sufficient growth was achieved by glucose and the cells entered the saturation stage, the feed was shifted to glycerol and lactose (as an inducer). Two major drawbacks were observed with this strategy in our case. First, the initial fast growth in CD media resulted in a loss/reduction in plasmid content (Figure 2A). This may lead to uneven distribution of plasmid in daughter cells, resulting in a rapid decrease in plasmid-bearing cells (unproductive cells). This may explain why, even though the process in CD media produced twice the biomass as in CM media, it still resulted in lower specific productivity and higher host cell impurities (Table 2). Second, a sudden transition from cell growth to the induction stage may disrupt cellular homeostasis, leading to the formation of inclusion bodies (IB) (Figure 1B). It has been reported [17] that factors such as high protein expression rates, environmental conditions or target protein properties are some of the many that dictate the formation of IB [18]. Since the chimeric protein expressed in this study is a multi-domain structure [19], its folding process may involve several intermediate phases. It may be plausible that a sudden transition from the growth to the induction phase, creating a mismatch between the expression rate and folding, may lead to misfolding/aggregation, eventually forming IB.

Additionally, *E. coli* cultures grown on carbon sources such as glucose require high oxygen since, in oxygen-restricted conditions, the metabolism of the microorganism is substantially altered, and by-products like organic acids, such as acetate and ethanol, may be formed [20]. Such conditions may not only adversely affect the growth but may also not be optimal for the expression of many recombinant proteins, and they often lead to the formation of inclusion bodies. Similarly, high temperature does help in high growth but may lead to variable productivity due to metabolic burden caused by uneven resource distribution [21,22].

On the contrary, in the process with CM media, the cells were allowed to grow slowly and adapt to milder conditions, with a stepwise increasing concentration of the inducer (galactose), resulting in higher plasmid retention by the cells (Figure 2B) and a substantial increase in specific productivity and reduced impurities (Table 2). Moreover, with the tighter control over nutrient supply and growth conditions (reducing oxygen concentration and temperature), a balance was achieved between cell growth and target protein induction, resulting in reduced metabolic burden and cellular stress, potentially leading to a higher proportion of properly folded, soluble protein in the cytoplasm (Figure 1B).

### 3.2. Structural Characteristics of the Chimeric Proteins Obtained from the CD and CM Media

As shown in the Figure 1C, the final purified chimeric proteins obtained from both media appear very similar in terms of their physical characteristics, such as native (Table 2) and subunit molecular mass. However, the tendency for misfolding, as indicated with the protein obtained from the CD media process, indicates that such propensity might affect the structural integrity of the protein and may interfere with its function or intended specific response. In order to assess the process-dependent structural differences, we probed and analyzed the secondary and tertiary folding patterns of the purified chimeric proteins obtained from both processes using Circular Dichroism (CD) spectroscopy [23].

**Figure 3:**
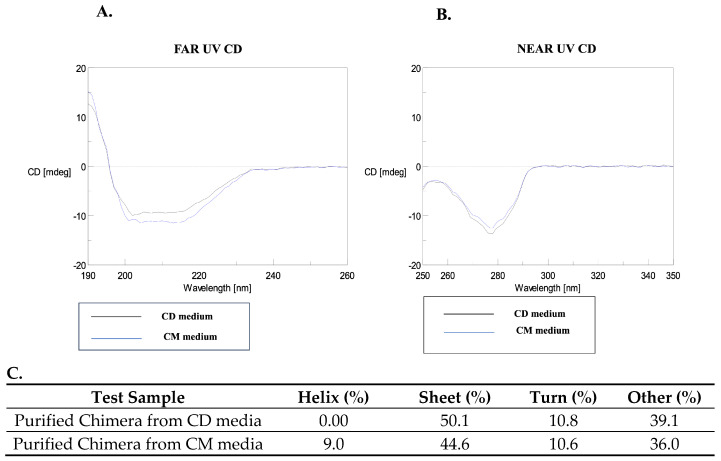
Circular dichroism spectroscopy of purified chimeric protein: Folding and secondary structure analysis of chimeric protein obtained from lactose-induced CD media (black) and galactose-induced autoinduction CM media (Blue). (**A**) Far-UV and (**B**) near-UV spectra. (**C**) The table below displays deconvoluted values obtained for each of the spectra.

For both the chimeras, the far UV-CD spectra (190–260 nm) displayed a single prominent negative band around 210–220 nm, indicating the characteristic β-sheet structures (Figure 3). However, the spectra for the proteins varied in their intensities and curvature, indicating some subtle structural changes in conformational organization. The near-UV CD spectra, which reflect the protein’s tertiary structure in terms of the spatial arrangement of aromatic amino acid side chains (tryptophan, tyrosine, phenylalanine) and disulfide bonds, showed a single prominent band at 275 nm, arising from a single tyrosine present in the chimeric proteins (Figure 3). The near-UV CD spectra showed no other discernible changes, indicating that the chimeras may have similar gross 3D structure. However, when the data was deconvoluted and analyzed using Specter Manager software (Version 2.15), the purified chimeras obtained from the respective processes showed substantial differences (Figure 3). The purified chimera from the CD media process primarily consisted of 50.1% β-sheet, 39.1% random structures, and nearly 0.0% ⍺-helical structures. In contrast, the protein obtained from the CM media process revealed 44.6% β-sheets, 36.0% random/disordered region and 9.0% ⍺-helical structure, indicating a marked difference in structure between the proteins obtained from each medium. Helix formation is known to provide a structural scaffold that guides protein through the early folding steps [24]; therefore, an increased protein helical content may indicate increased stability of a protein as it moves from an unfolded to a folded state. A previous report on the structure of the full-length fHbp by NMR [19] established the distribution of protective epitopes and possible localization of the factor H binding site. The results of the study revealed that the full-length fHbp adopts a solution structure consisting of two domains, an N-terminal (8–136 residues) domain and a C-terminal (141–255) domain, each composed of 10 and 8 antiparallel beta strands, respectively. Each domain has two short alpha helix stretches, an N-terminal (16–21), which helps anchor N-terminal beta strands to the rest of the protein, and a very short (141–143) stretch that connects the two fHbp domains and part of a loop (146–149), which was identified to be involved in the formation of bactericidal epitope.

The presence of alpha helical content in the CM media process-derived protein thus suggests improved secondary structure formation and stability, which may relate to the possible native conformation of fHbp. Thus, it may be possible that the presence of helical content could be the apparent factor responsible for the high proportion of properly folded, soluble protein observed in the CM media process (Figure 1B) and may also be critical in the correct orientation of protective epitopes. In contrast, in the case of the CD media-derived protein, the absence of a helical structure, which is crucial for stabilizing the folding intermediates during protein folding, may inadvertently expose the hydrophobic residues to the aqueous environment, promoting aggregation and resulting in IB formation.

Together, the data from CD spectroscopy suggest that the composition of the growth media and the process parameters had significant influence on the folding efficiency and conformational integrity of both of the chimeric proteins. The nutrient-rich CM media provided a sufficiently conducive environment for promoting native protein-like structure, whereas CD media limited these favorable settings, resulting in subtle variations that may influence the consistency of product quality.

### 3.3. In Vitro and In Vivo Immunological Characteristics of Chimeric Protein Obtained from CD and CM Media

Structural consistency of a recombinant protein is not only an important functional quality attribute but also has direct implications on immunological outcomes. Therefore, proper folding is as critical for protein stability as it is for immunogenicity. An incorrect conformational fold can lead to an epitope orientation that is less likely to elicit the desired immune response. Several reports suggest the significance of recombinant protein conformational stability for immunogenicity and functional potential in vaccines [25].

Previous studies with the fHbp-PorA chimera demonstrated that this strategy of fusion construct maintains epitope accessibility and supports recognition by bactericidal monoclonal antibodies. This resulted in robust serum bactericidal activity against both clinical isolates and engineered isogenic mutants, leading simultaneously to differentiation of the immune response directed towards each component of the chimeric protein [11].

**Figure 4:**
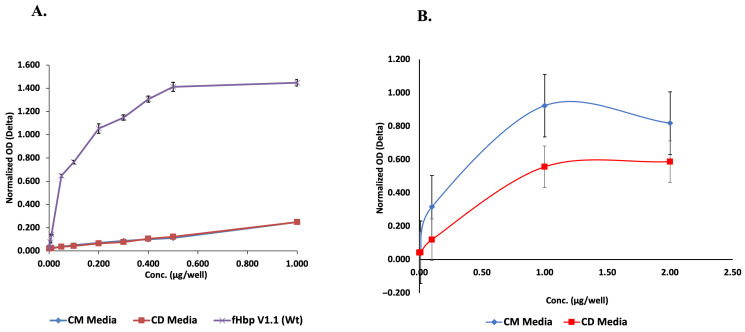
Comparative analysis of (**A**) fHbp- and (**B**) PorA-specific ELISA using monoclonal antibodies targeting the respective portions of the chimeric protein obtained from lactose-induced CD media vs. galactose-induced autoinduction media. (The represented values are the means of three experiments).

However, as revealed by the above-mentioned structural analysis, the chimera from the CM media process exhibited better folding kinetics over the chimera from the CD media process. Therefore, it was intriguing to determine if both also differed in terms of their immunological features. In order to evaluate and verify the antigenic structural integrity and epitope exposure, we employed the ELISA technique and probed each protein with a monoclonal antibody that identified an epitope, specific either to the fH binding site of fHbp or the PorA loop of the chimeric protein. As shown in Figure 4, the specific interaction with the fHbp scaffold for both proteins completely overlap, indicating the structural similarity within this part of the chimeric protein. However, with respect to specific interaction with the PorA loop, the chimera from the CM media showed prominent and higher intensity response compared to chimera from the CD media. This marked difference in epitope projection and recognition clearly indicates a change in the conformational orientation of the two proteins.

fHbp epitopes have been extensively mapped using a variety of methods, such as phage display, cryo-electron microscopy, and monoclonal antibodies [26]. All these studies reveal that the fHbp molecule has multiple bactericidal conformational epitopes along both the C and N terminal domains. Additionally, it was proposed that fHbp-mediated immune response is synergistically coordinated by binding to these epitopes [27]. Therefore, optimal distance between these epitopes can not only promote activation of a complement system but also enable binding to two overlapping epitopes, inducing bactericidal activity in strains expressing low levels of fHbp. Thus, the ELISA result further supports the view that the protein folding environment significantly impacts the structural integrity of both the chimeric proteins and in turn may influence the epitope binding properties.

In order to gain further insight into the implications of altered conformational orientation of the two chimeric proteins on *in vivo* immunogenicity, we immunized rabbits with chimeric proteins obtained from both processes. We then evaluated the immune response by measuring the human complement-dependent serum bactericidal activity (hSBA) titers against two homologous (fHbp 1.1-PorA 1.16 (M08240157), fHbp 1.4-PorA 1.4 (M17240832) and two heterologous (fHbp 3.45-PorA 1.14 (M17240156), fHbp 2.19-PorA 1.9 (M16240272)) *Neisseria meningitidis* serogroup B clinical isolates. The hSBA titer of ≥1:4 is the most accepted correlate of protection against the meningococcal serogroup. The hSBA titers were measured in sera collected on Day 0 (pre-immune), Day 28 (post-primary booster)and Day 36 (secondary booster).

**Figure 5:**
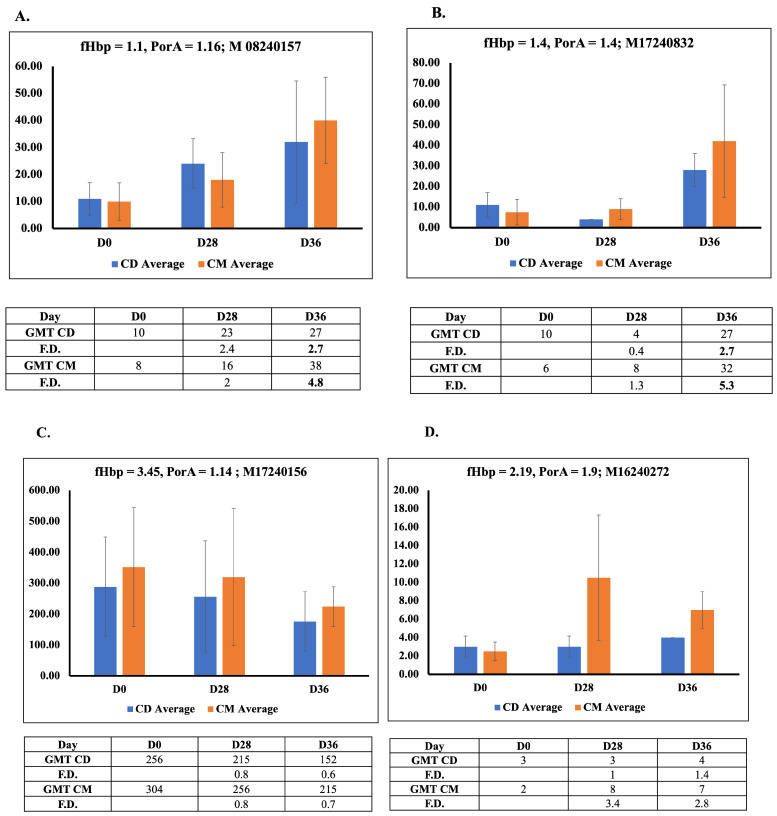

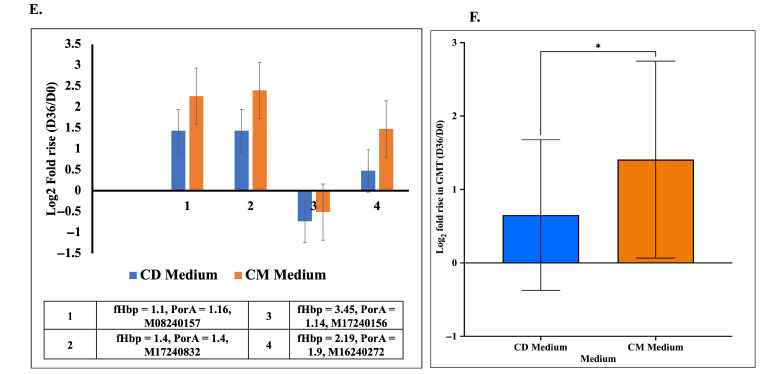
Immunogenicity of chimeric proteins obtained from lactose-induced CD media vs. galactose-induced autoinduction media in rabbits (**A**–**F**). Each panel represents the geometric mean hSBA titers obtained on three sampling days, tested against two homologous (**A**,**B**) and two heterologous (**C**,**D**) Meningococcal B clinical isolates. (**E**) Comparison of antibody responses induced by chimeric fHbp-PorA protein produced in CD and CM media. Log_2_-transformed geometric mean titer (GMT) fold rise (D36/D0) is shown for individual clinical isolates. (**F**) Mean log_2_ GMT fold rise (D36/D0) across all strains. Error bars represent standard deviations among the tested sample data set. Paired *t*-test analysis was used to compare SBA titers of CD and CM media (* *p* ≤ 0.05).

As shown in Figure 5 all the groups exhibited low hSBA titers at the baseline; however, following immunization, both chimeras induced noteworthy increases in titer levels by Day 28 and Day 36, especially against the homologous strains. In contrast, titers against the heterologous strains remained low, indicating strain-specific immune response. This emphasizes the need for a multivalent/combination vaccine to achieve broad-spectrum protection and agrees with previously reported observations of a high degree of variability in fHbp that prevents cross-protection among three variant isolates.

Interestingly, when the geometric mean titers (GMTs) and fold increases in immune response for the chimera from both media were compared, the CM media-derived formulation clearly and consistently produced acceptable (≥4) GMTs across homologous strains (Figure 5). This suggests that the antigenic chimeric protein produced in the CM media process may possess improved conformational integrity and necessary epitopes that enhance immune recognition. Furthermore, the increase in titer after the booster dose demonstrates the elicitation of memory B cell response, a feature for long-term protection. The sustained adequate fold difference in hSBA titers, particularly with the CM media-derived protein, thus supports the potential for galactose-induced autoinduction as a promising process to prepare these antigen vaccine components.

Overall, these results evidently support and align with the above-mentioned findings that the choice of media and expression conditions significantly impacts the structural integrity of the expressed recombinant proteins arising from subtle differences in protein folding, solubility, and the presence of host-cell contaminants (like, HCD, HCP, LPS). The media itself does not directly determine immunogenicity, but it plays a critical role in determining the quality of the chimeric protein and the subsequent magnitude of the immune response it elicits.

## 4. Conclusions

Autoinduction in *E. coli* is a well-established system for recombinant protein production, which basically relies on decoupling cell growth and protein expression in order to effectively use the resources and reduce the host cell burden. However, this study establishes that it may not be an unequivocal or favorable condition applicable to all recombinant proteins. The results shown in this study clearly demonstrate that, for a complicated multi-domain protein such as fHbp-PorA chimera, synchronized growth and protein expression achieved using CM media and the inducer galactose is the most effective, efficient and consistent way to produce this promising chimeric protein. The data reaffirms our previous report that galactose-induced autoinduction not only helps achieve high cell densities and specific productivity but also obtains the protein with the right quality attributes for its intended immunological application as a vaccine. As we further take this concept of chimeric-based protein into preclinical and clinical trials, this process guarantees product safety, low process cost and regulatory compliance, as it does not use or need toxic chemicals, such as IPTG, antibiotics or animal-derived products. However, owing to its very specific immune reactivity to homologous clinical isolates, this study also discreetly indicates the use of a combination of these chimeric proteins to increase the spectrum of vaccine coverage.

## Figures and Tables

**Table 1:** Rabbit immunization study design to test the immunogenicity of chimeric proteins obtained from lactose-induced CD media vs. galactose-induced autoinduction media.

S. No.	Media	Test Sample	Dose (μg)/inj	Animals Injected	Route	Injection Vol.	Day of Dosing	Sera Collection Day		
								Day 0	Day 28	Day 36
1	CD	CD media formulation	30	4	IM	500	1, 14, 28	4	4	4
2	CM	CM media formulation	30	4	IM	500	1, 14, 28	4	4	4
Total Rabbits				8				8	8	8
Total Sera Samples								24		

**Table 2:** Comparative evaluation of specific productivity, impurity profile and purity of the chimeric protein between lactose-induced CD media vs. galactose-induced autoinduction CM media. (The represented values are the means of three experiments).

Process Details	CD Media Process	CM Media Process
Total Cell mass	200 g/L	80 g/L
Tagged protein (mg/gram of cells)	13.93	23.84
Final fHbp protein yield (%) from 75 Gm of tagged protein	9.20%	16.32%
SEC-HPLC	Monomeric peak purity of 95.52%	Monomeric peak purity of 98.60%
Mol. Wt. SDS-PAGE	27.8 Kda	27.8 Kda
Host cell protein (HCP)	60.3 ng/mg	12.6 ng/mg
Host cell DNA (HCD)	0.56 pg/mg	0.06 pg/mg
TEV protease residual	<1.00 ng/mg	<0.592 ng/mg
Bacterial endotoxin	182.89 EU/mL 133.50 EU/mg	14.255 EU/mL 6.392 EU/mg

## Data Availability

The original contributions presented in this study are included in the article. Further inquiries can be directed to the corresponding author.

## Supplementary Materials

The following supporting information can be downloaded at: https://www.mdpi.com/article/10.3390/vaccines14050382/s1, The original SDS-PAGE figures.
