# Supplementary material for: Impact of Media and Induction Strategy on Physicochemical Characteristics and Immunogenicity of Recombinant fHbp-PorA Chimeric Protein: A Promising Meningococcal B Vaccine Candidate Produced in Escherichia coli
Source: Vaccines (Basel). 2026 Apr 24;14(5):382. doi: 10.3390/vaccines14050382 (PMC13211582; doi:10.3390/vaccines14050382)

Supplementary information for "Impact of Media and Induction Strategy on Physicochemical Characteristics and Immunogenicity of Recombinant fHbp-PorA Chimeric Protein: A Promising Meningococcal B Vaccine Candidate Produced in *Escherichia coli*"

Authors:

Annamraju Aswini [1,2], Annamraju D. Sarma [1], Ashish B. Deshpande [1], Yogesh C. Padwal [1], Vinay V. Gavade [1], Sambhaji S. Pisal [1] and Selvan Ravindran [2,*]

1 Serum Institute of India Pvt. Ltd., Hadapsar, Pune 411028, India

2 Symbiosis School of Biological Sciences (SSBS), Faculty of Medical and Health Sciences, Symbiosis International (Deemed University), Lavale, Pune 412115, India

* Correspondence: selvan.ravindran@ssbs.edu.in or selvan_ravindran@yahoo.com; Tel.: +91-9673555665 or +91-9049480567

(**B**) Comparative SDS-PAGE analysis of expressed target protein in CD media using lactose induc-tion (lane 1: insoluble fraction; lane 2: soluble fraction) and CM media with galactose autoinduc-tion (lane 3: insoluble fraction; lane 4: soluble fraction). (**C**) Comparative SDS-PAGE analysis of purified tagged chimeric protein (lanes 1 and 3) and final chimeric protein without tag (lanes 2 and 4) from CD and CM media, respectively

The original SDS-PAGE of Figure 1B

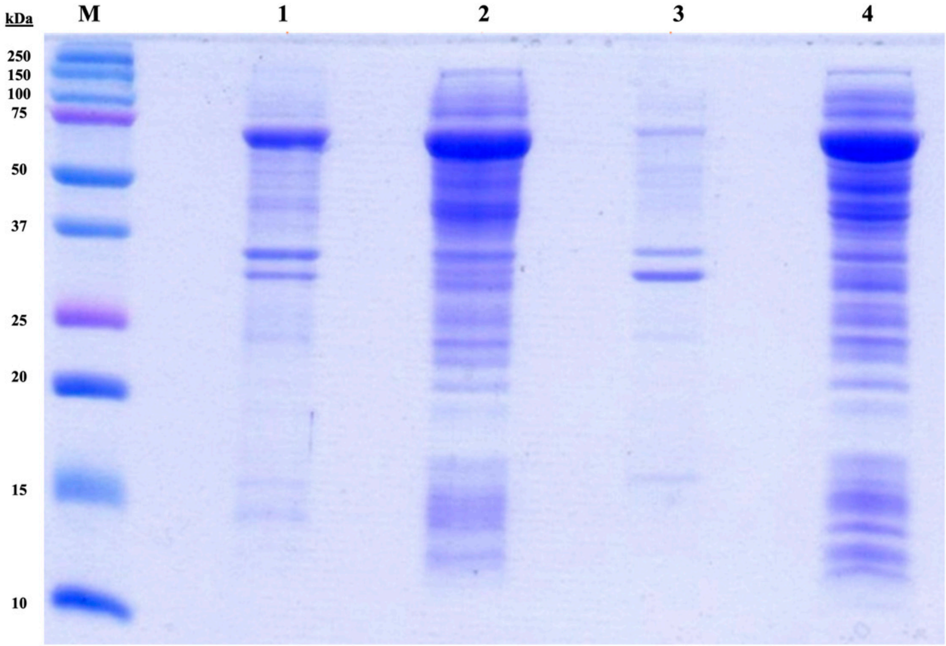

The original SDS-PAGE Figure 1C

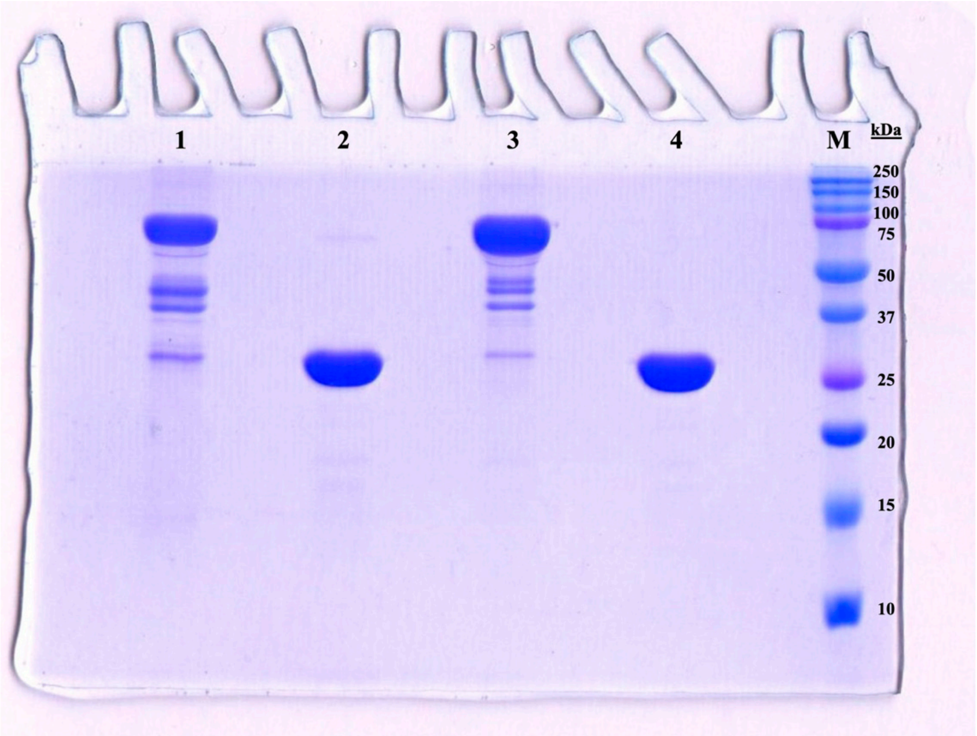

Supplement: Supplementary file 1 [file vaccines-14-00382-s001.zip › vaccines-4135174-supplementary.pdf]
